# [*N*-(2-Hydroxy­ethyl)ethyl­enediamine]oxalatocopper(II)

**DOI:** 10.1107/S1600536809040264

**Published:** 2009-10-10

**Authors:** Hümeyra Paşaoğlu, Gökhan Kaştaş, Okan Z. Yeşilel, M. Hakkı Yıldırım

**Affiliations:** a Department of Physics, Faculty of Arts and Sciences, Ondokuz Mayıs University, Kurupelit TR-55139, Samsun, Turkey; b Department of Chemistry, Faculty of Arts and Sciences, Eskişehir Osmangazi University, TR-26480 Eskişehir, Turkey

## Abstract

In the title mononuclear copper(II) compound, [Cu(C_2_O_4_)(C_4_H_12_N_2_O)], the Cu^II^ ion has a slightly distorted square-pyramidal geometry, with a tridentate *N*-(2-hydroxy­ethyl)ethyl­enediamine (HydEt-en) and a bidentate oxalate (ox) ligand. The N atoms of the HydEt-en ligand and the O atoms of ox ligand form the basal plane, while the O atom of the ethanol group of the HydEt-en ligand is located in the axial position. The complex mol­ecules participate in a supra­molecular assembly through N—H⋯O and O—H⋯O hydrogen bonds between HydEt-en and ox ligands.

## Related literature

For general background to the HydEt-en ligand, see: Karadağ *et al.* (2004 ▶, 2005 ▶); Paşaoğlu *et al.* (2005 ▶). For transition metal complexes of oxalate, see: Scott *et al.* (1973 ▶); Xia *et al.* (2004 ▶); Yılmaz *et al.* (2003 ▶); Youngme *et al.* (2003 ▶). For graph-set notation, see: Bernstein *et al.* (1995 ▶).
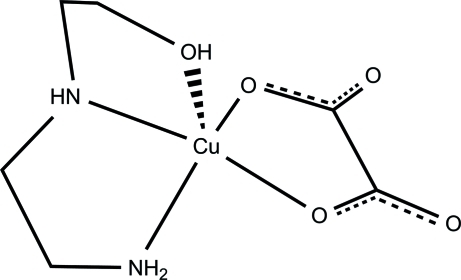


## Experimental

### 

#### Crystal data

[Cu(C_2_O_4_)(C_4_H_12_N_2_O)]
*M* *_r_* = 255.72
Orthorhombic, 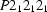

*a* = 7.9766 (5) Å
*b* = 8.7263 (4) Å
*c* = 13.0191 (7) Å
*V* = 906.21 (9) Å^3^
*Z* = 4
Mo *K*α radiation
μ = 2.41 mm^−1^
*T* = 296 K
0.52 × 0.42 × 0.23 mm

#### Data collection

Stoe IPDS-II diffractometer
Absorption correction: integration (*X-RED32*; Stoe & Cie, 2002 ▶) *T*_min_ = 0.570, *T*_max_ = 0.781
10045 measured reflections
1868 independent reflections
1780 reflections with *I* > 2σ(*I*)
*R*_int_ = 0.063

#### Refinement

*R*[*F* ^2^ > 2σ(*F* ^2^)] = 0.029
*wR*(*F* ^2^) = 0.065
*S* = 1.08
1868 reflections
143 parameters
H atoms treated by a mixture of independent and constrained refinement
Δρ_max_ = 0.22 e Å^−3^
Δρ_min_ = −1.30 e Å^−3^
Absolute structure: Flack (1983 ▶), 797 Friedel pairs
Flack parameter: 0.017 (17)

### 

Data collection: *X-AREA* (Stoe & Cie, 2002 ▶); cell refinement: *X-AREA*; data reduction: *X-RED32* (Stoe & Cie, 2002 ▶); program(s) used to solve structure: *SHELXS97* (Sheldrick, 2008 ▶); program(s) used to refine structure: *SHELXL97* (Sheldrick, 2008 ▶); molecular graphics: *ORTEP-3* (Farrugia, 1997 ▶); software used to prepare material for publication: *WinGX* (Farrugia, 1999 ▶).

## Supplementary Material

Crystal structure: contains datablocks global, I. DOI: 10.1107/S1600536809040264/ci2929sup1.cif

Structure factors: contains datablocks I. DOI: 10.1107/S1600536809040264/ci2929Isup2.hkl

Additional supplementary materials:  crystallographic information; 3D view; checkCIF report

## Figures and Tables

**Table 1:** Selected bond lengths (Å)

Cu1—O1	1.9505 (13)
Cu1—O4	1.9625 (14)
Cu1—N2	1.9717 (18)
Cu1—N1	2.0066 (18)
Cu1—O5	2.4174 (16)

**Table 2:** Hydrogen-bond geometry (Å, °)

*D*—H⋯*A*	*D*—H	H⋯*A*	*D*⋯*A*	*D*—H⋯*A*
N1—H1⋯O4^i^	0.85 (3)	2.17 (3)	3.015 (2)	173 (2)
N2—H2*A*⋯O1^ii^	0.94 (3)	2.02 (3)	2.936 (2)	165 (2)
N2—H2*B*⋯O2^iii^	0.91 (3)	2.02 (3)	2.909 (2)	168 (3)
O5—H5⋯O3^iv^	0.72 (3)	2.06 (3)	2.774 (3)	171 (3)

Symmetry codes: (i) 
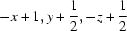
; (ii) 
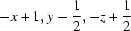
; (iii) 
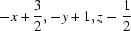
; (iv) 
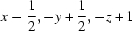
.

**Fig. 1.**
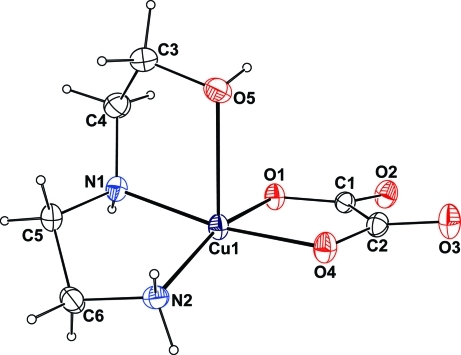
The molecular structure of [Cu(HydEt-en)(ox)] with atom-labeling scheme. Displacement ellipsoids are drawn at the 30% probability level for the non hydrogen atoms.

**Fig. 2.**
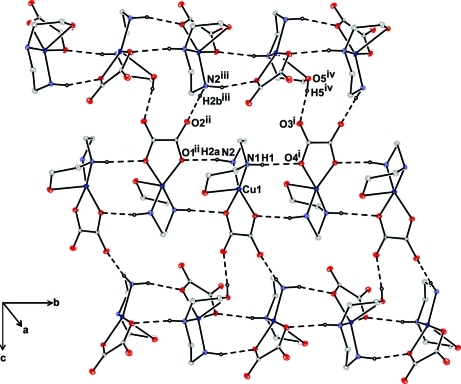
The assembly of polymeric chains of [Cu(HydEt-en)(ox)] into a two-dimensional layer by N—H···O and O—H···O hydrogen bonds. H atoms not involved in hydrogen bondings have been omitted for clarity. Symmetry codes are as given in Table 2

**Fig. 3.**
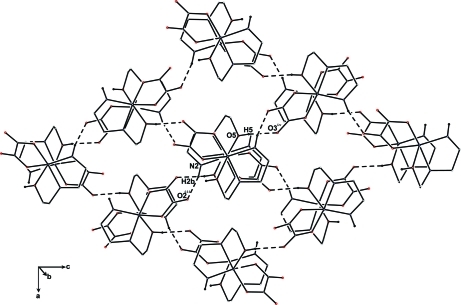
The supramolecular network of [Cu(HydEt-en)(ox)] projected onto (010). All H atoms except H2b and H5 have been omitted for clarity. Symmetry codes are as given in Table 2.

## supplementary crystallographic information

### Comment

As part of our ongoing research on the preparation and characterization of mixed ligand metal complexes of HydEt-en we report here the synthesis and X-ray analysis of a mononuclear copper(II) complex, [Cu(HydEt-en)(ox)]. This study is an example of the construction of a supramolecular assembly based on hydrogen bonds in mixed-ligand metal complexes.

In title compound, the HydEt-en ligand chelates through its two N atoms and the O atom of the hydroxyl group. The square-pyramidal coordination shell consists of three five-membered chelate rings (Fig. 1) *viz*. A (Cu1/O1/C1/C2/O4), B (Cu1/N1/C5/C6/N2) and C (Cu1/O5/C3/C4/N1). The mean plane through ring C is perpendicular to that through the ring A, with a dihedral angle of 89.27 (5)°. The bite angles of rings B and C are 86.36 (7)° and 78.65 (7)°, respectively.

The complex participates in a supramolecular assembly through N—H···O and O—H···O hydrogen bonds between HydEt-en and oxalate ligands. The HydEt-en ligand is involved in hydrogen bonds through its amino, imino and hydroxyl groups. In the crystal structure (Fig. 2), N1—H1···O4^i^ and N2—H2A···O1^ii^ (Table 2) hydrogen bonds constitute a polymeric chain parallel to the [010], giving rise to C(4) chain and *R*_2_^2^(8) (Bernstein *et al.*, 1995) rings. These polymeric chains are inter-connected to each other by N2—H2B···O2^iii^ and O5—H5···O3^iv^ hydrogen bonds extending through the *ac* plane, resulting in a three-dimensional supramolecular network as illustrated in Fig. 3.

### Experimental

The HydEt-en ligand (0.12 g, 2 mmol) was added dropwise to a solution of Cu(ox).0.5H_2_O (0.48 g, 3.0 mmol) in pyridine-water (1:2, 30 ml) at 50° C. The resulting solution was stirred for 1 h at 50° C and then filtered. The reaction mixture was then slowly cooled to room temperature. Violet crystals suitable for X-ray diffraction analysis were obtained after a few days and were washed with 5 ml of ethanol and dried in air.

### Refinement

All H atoms involved in hydrogen bondings were located in a difference Fourier map and their positional and U_iso_ parameters were refined. The remaining H atoms were placed in calculated positions and constrained to ride on their parents atoms, with C-H = 0.97 Å and *U*_iso_(H) = 1.2*U*_eq_(C).

### Crystal data

**Table:**

[Cu(C_2_O_4_)(C_4_H_12_N_2_O)]	*F*(000) = 524
*M**_r_* = 255.72	*D*_x_ = 1.874 Mg m^−^^3^
Orthorhombic, *P*2_1_2_1_2_1_	Mo *K*α radiation, λ = 0.71073 Å
Hall symbol: P 2ac 2ab	Cell parameters from 10045 reflections
*a* = 7.9766 (5) Å	θ = 1.6–28.0°
*b* = 8.7263 (4) Å	µ = 2.41 mm^−^^1^
*c* = 13.0191 (7) Å	*T* = 296 K
*V* = 906.21 (9) Å^3^	Prism, violet
*Z* = 4	0.52 × 0.42 × 0.23 mm

### Data collection

**Table:**

Stoe IPDS-II diffractometer	1868 independent reflections
Radiation source: fine-focus sealed tube	1780 reflections with *I* > 2σ(*I*)
plane graphite	*R*_int_ = 0.063
Detector resolution: 6.67 pixels mm^-1^	θ_max_ = 26.5°, θ_min_ = 2.8°
ω scans	*h* = −10→10
Absorption correction: integration (*X-RED32*; Stoe & Cie, 2002)	*k* = −10→10
*T*_min_ = 0.570, *T*_max_ = 0.781	*l* = −15→16
10045 measured reflections	

### Refinement

**Table:**

Refinement on *F*^2^	Secondary atom site location: difference Fourier map
Least-squares matrix: full	Hydrogen site location: inferred from neighbouring sites
*R*[*F*^2^ > 2σ(*F*^2^)] = 0.029	H atoms treated by a mixture of independent and constrained refinement
*wR*(*F*^2^) = 0.065	*w* = 1/[σ^2^(*F*_o_^2^) + (0.0427*P*)^2^] where *P* = (*F*_o_^2^ + 2*F*_c_^2^)/3
*S* = 1.08	(Δ/σ)_max_ = 0.001
1868 reflections	Δρ_max_ = 0.22 e Å^−^^3^
143 parameters	Δρ_min_ = −1.30 e Å^−^^3^
0 restraints	Absolute structure: Flack (1983), 797 Friedel pairs
Primary atom site location: structure-invariant direct methods	Flack parameter: 0.017 (17)

### Special details

**Table:**

Geometry. All e.s.d.'s (except the e.s.d. in the dihedral angle between two l.s. planes) are estimated using the full covariance matrix. The cell e.s.d.'s are taken into account individually in the estimation of e.s.d.'s in distances, angles and torsion angles; correlations between e.s.d.'s in cell parameters are only used when they are defined by crystal symmetry. An approximate (isotropic) treatment of cell e.s.d.'s is used for estimating e.s.d.'s involving l.s. planes.
Refinement. Refinement of *F*^2^ against ALL reflections. The weighted *R*-factor *wR* and goodness of fit *S* are based on *F*^2^, conventional *R*-factors *R* are based on *F*, with *F* set to zero for negative *F*^2^. The threshold expression of *F*^2^ > σ(*F*^2^) is used only for calculating *R*-factors(gt) *etc*. and is not relevant to the choice of reflections for refinement. *R*-factors based on *F*^2^ are statistically about twice as large as those based on *F*, and *R*- factors based on ALL data will be even larger.

### Fractional atomic coordinates and isotropic or equivalent isotropic displacement parameters (Å^2^)

**Table:**

	*x*	*y*	*z*	*U*_iso_*/*U*_eq_	
C1	0.6054 (2)	0.4652 (2)	0.44437 (15)	0.0242 (4)	
C2	0.7217 (3)	0.3322 (2)	0.41059 (16)	0.0274 (4)	
C3	0.1649 (3)	0.2157 (3)	0.28011 (18)	0.0389 (5)	
H3A	0.0821	0.1836	0.3302	0.047*	
H3B	0.1451	0.1589	0.2172	0.047*	
C4	0.1448 (3)	0.3853 (2)	0.25922 (18)	0.0374 (5)	
H4A	0.0389	0.4029	0.2244	0.045*	
H4B	0.1422	0.4404	0.3239	0.045*	
C5	0.2752 (3)	0.3933 (3)	0.08732 (16)	0.0334 (4)	
H5A	0.1974	0.4569	0.0492	0.040*	
H5B	0.2357	0.2883	0.0845	0.040*	
C6	0.4476 (3)	0.4039 (3)	0.04004 (16)	0.0339 (4)	
H6A	0.4487	0.3530	−0.0262	0.041*	
H6B	0.4780	0.5105	0.0299	0.041*	
Cu1	0.50643 (3)	0.38250 (2)	0.252350 (16)	0.02606 (11)	
N1	0.2830 (2)	0.44501 (19)	0.19527 (13)	0.0271 (4)	
N2	0.5680 (2)	0.3297 (2)	0.11000 (14)	0.0282 (4)	
O1	0.48949 (18)	0.49776 (14)	0.38029 (11)	0.0288 (3)	
O2	0.6303 (2)	0.53159 (18)	0.52577 (12)	0.0364 (4)	
O3	0.8216 (2)	0.27578 (18)	0.47136 (13)	0.0408 (4)	
O4	0.70385 (19)	0.28980 (16)	0.31740 (11)	0.0328 (3)	
O5	0.3275 (2)	0.18117 (19)	0.31756 (14)	0.0351 (3)	
H1	0.280 (3)	0.542 (3)	0.1959 (18)	0.028 (6)*	
H2A	0.559 (3)	0.223 (3)	0.102 (2)	0.038 (7)*	
H2B	0.668 (4)	0.371 (4)	0.093 (2)	0.052 (8)*	
H5	0.319 (3)	0.186 (3)	0.373 (2)	0.030 (7)*	

### Atomic displacement parameters (Å^2^)

**Table:**

	*U*^11^	*U*^22^	*U*^33^	*U*^12^	*U*^13^	*U*^23^
C1	0.0248 (9)	0.0247 (8)	0.0231 (9)	−0.0019 (7)	−0.0017 (8)	−0.0006 (7)
C2	0.0259 (9)	0.0240 (8)	0.0323 (10)	0.0001 (8)	−0.0045 (8)	0.0010 (8)
C3	0.0350 (12)	0.0418 (11)	0.0400 (11)	−0.0101 (10)	−0.0008 (10)	0.0076 (9)
C4	0.0307 (10)	0.0416 (11)	0.0399 (13)	0.0025 (8)	0.0031 (9)	0.0028 (11)
C5	0.0340 (10)	0.0397 (10)	0.0266 (10)	−0.0020 (10)	−0.0076 (8)	0.0005 (9)
C6	0.0419 (11)	0.0360 (9)	0.0238 (10)	−0.0051 (9)	−0.0019 (9)	0.0035 (8)
Cu1	0.02695 (16)	0.02892 (15)	0.02230 (18)	0.00454 (9)	−0.00390 (9)	−0.00309 (8)
N1	0.0286 (8)	0.0228 (8)	0.0299 (9)	0.0020 (6)	−0.0050 (7)	−0.0012 (6)
N2	0.0280 (9)	0.0289 (8)	0.0276 (9)	−0.0039 (7)	0.0030 (7)	−0.0019 (7)
O1	0.0315 (7)	0.0291 (6)	0.0258 (6)	0.0055 (6)	−0.0055 (6)	−0.0039 (5)
O2	0.0366 (9)	0.0409 (7)	0.0316 (8)	0.0008 (7)	−0.0068 (7)	−0.0103 (7)
O3	0.0415 (9)	0.0449 (8)	0.0359 (8)	0.0151 (7)	−0.0119 (7)	−0.0018 (7)
O4	0.0321 (7)	0.0363 (8)	0.0299 (7)	0.0109 (6)	−0.0046 (7)	−0.0068 (6)
O5	0.0370 (8)	0.0366 (8)	0.0318 (8)	−0.0002 (7)	0.0049 (7)	0.0061 (7)

### Geometric parameters (Å, °)

**Table:**

C1—O2	1.224 (2)	C5—H5A	0.97
C1—O1	1.277 (2)	C5—H5B	0.97
C1—C2	1.549 (3)	C6—N2	1.473 (3)
C2—O3	1.226 (3)	C6—H6A	0.97
C2—O4	1.276 (3)	C6—H6B	0.97
C3—O5	1.418 (3)	Cu1—O1	1.9505 (13)
C3—C4	1.514 (3)	Cu1—O4	1.9625 (14)
C3—H3A	0.97	Cu1—N2	1.9717 (18)
C3—H3B	0.97	Cu1—N1	2.0066 (18)
C4—N1	1.477 (3)	Cu1—O5	2.4174 (16)
C4—H4A	0.97	N1—H1	0.85 (3)
C4—H4B	0.97	N2—H2A	0.94 (3)
C5—N1	1.477 (3)	N2—H2B	0.91 (3)
C5—C6	1.509 (3)	O5—H5	0.72 (3)
			
O2—C1—O1	125.27 (18)	H6A—C6—H6B	108.4
O2—C1—C2	120.22 (17)	O1—Cu1—O4	84.24 (6)
O1—C1—C2	114.50 (16)	O1—Cu1—N2	160.11 (7)
O3—C2—O4	124.72 (19)	O4—Cu1—N2	96.29 (7)
O3—C2—C1	120.44 (18)	O1—Cu1—N1	96.58 (6)
O4—C2—C1	114.83 (16)	O4—Cu1—N1	169.93 (7)
O5—C3—C4	111.52 (19)	N2—Cu1—N1	86.36 (7)
O5—C3—H3A	109.3	O1—Cu1—O5	91.94 (6)
C4—C3—H3A	109.3	O4—Cu1—O5	91.30 (6)
O5—C3—H3B	109.3	N2—Cu1—O5	107.90 (7)
C4—C3—H3B	109.3	N1—Cu1—O5	78.65 (7)
H3A—C3—H3B	108.0	C4—N1—C5	113.41 (17)
N1—C4—C3	111.52 (19)	C4—N1—Cu1	111.01 (13)
N1—C4—H4A	109.3	C5—N1—Cu1	107.84 (13)
C3—C4—H4A	109.3	C4—N1—H1	109.0 (17)
N1—C4—H4B	109.3	C5—N1—H1	108.3 (16)
C3—C4—H4B	109.3	Cu1—N1—H1	107.1 (16)
H4A—C4—H4B	108.0	C6—N2—Cu1	108.44 (13)
N1—C5—C6	109.33 (17)	C6—N2—H2A	108.3 (16)
N1—C5—H5A	109.8	Cu1—N2—H2A	108.5 (16)
C6—C5—H5A	109.8	C6—N2—H2B	104 (2)
N1—C5—H5B	109.8	Cu1—N2—H2B	111 (2)
C6—C5—H5B	109.8	H2A—N2—H2B	116 (3)
H5A—C5—H5B	108.3	C1—O1—Cu1	113.13 (11)
N2—C6—C5	108.33 (17)	C2—O4—Cu1	112.35 (12)
N2—C6—H6A	110.0	C3—O5—Cu1	105.36 (12)
C5—C6—H6A	110.0	C3—O5—H5	104 (2)
N2—C6—H6B	110.0	Cu1—O5—H5	112 (2)
C5—C6—H6B	110.0		
			
O2—C1—C2—O3	11.9 (3)	O4—Cu1—N2—C6	−173.13 (13)
O1—C1—C2—O3	−169.41 (19)	N1—Cu1—N2—C6	16.62 (13)
O2—C1—C2—O4	−167.92 (19)	O5—Cu1—N2—C6	93.46 (14)
O1—C1—C2—O4	10.8 (2)	O2—C1—O1—Cu1	173.14 (16)
O5—C3—C4—N1	50.6 (3)	C2—C1—O1—Cu1	−5.5 (2)
N1—C5—C6—N2	49.2 (2)	O4—Cu1—O1—C1	0.32 (13)
C3—C4—N1—C5	71.6 (2)	N2—Cu1—O1—C1	−92.3 (2)
C3—C4—N1—Cu1	−50.0 (2)	N1—Cu1—O1—C1	170.23 (14)
C6—C5—N1—C4	−157.56 (17)	O5—Cu1—O1—C1	91.43 (13)
C6—C5—N1—Cu1	−34.2 (2)	O3—C2—O4—Cu1	170.12 (18)
O1—Cu1—N1—C4	−64.96 (14)	C1—C2—O4—Cu1	−10.1 (2)
O4—Cu1—N1—C4	29.1 (4)	O1—Cu1—O4—C2	5.92 (14)
N2—Cu1—N1—C4	134.79 (14)	N2—Cu1—O4—C2	165.93 (14)
O5—Cu1—N1—C4	25.71 (13)	N1—Cu1—O4—C2	−89.3 (4)
O1—Cu1—N1—C5	170.23 (13)	O5—Cu1—O4—C2	−85.90 (14)
O4—Cu1—N1—C5	−95.7 (4)	C4—C3—O5—Cu1	−25.7 (2)
N2—Cu1—N1—C5	9.98 (14)	O1—Cu1—O5—C3	96.66 (14)
O5—Cu1—N1—C5	−99.10 (14)	O4—Cu1—O5—C3	−179.06 (14)
C5—C6—N2—Cu1	−39.46 (19)	N2—Cu1—O5—C3	−82.02 (15)
O1—Cu1—N2—C6	−82.7 (2)	N1—Cu1—O5—C3	0.34 (14)

### Hydrogen-bond geometry (Å, °)

**Table:**

*D*—H···*A*	*D*—H	H···*A*	*D*···*A*	*D*—H···*A*
N1—H1···O4^i^	0.85 (3)	2.17 (3)	3.015 (2)	173 (2)
N2—H2A···O1^ii^	0.94 (3)	2.02 (3)	2.936 (2)	165 (2)
N2—H2B···O2^iii^	0.91 (3)	2.02 (3)	2.909 (2)	168 (3)
O5—H5···O3^iv^	0.72 (3)	2.06 (3)	2.774 (3)	171 (3)

Symmetry codes: (i) −*x*+1, *y*+1/2, −*z*+1/2; (ii) −*x*+1, *y*−1/2, −*z*+1/2; (iii) −*x*+3/2, −*y*+1, *z*−1/2; (iv) *x*−1/2, −*y*+1/2, −*z*+1.
